# *Saprochete capitata*: Emerging Infections from Uncommon Microorganisms in Hematological Diseases

**DOI:** 10.3390/hematolrep14020011

**Published:** 2022-03-24

**Authors:** Andrea Duminuco, Calogero Vetro, Cinzia Maugeri, Elisa Mauro, Giuseppe A. M. Palumbo, Marina S. Parisi, Benedetta Esposito, Giuseppe Giuliano, Alessandra Romano, Francesco Di Raimondo

**Affiliations:** 1 Post Graduation School of Hematology, University of Catania, A.O.U. “Policlinico-San Marco”, Via S. Sofia 78, 95123 Catania, Italy; benedetta.esposito89@gmail.com; 2 Division of Hematology, A.O.U. “Policlinico-San Marco”, Via S. Sofia 78, 95123 Catania, Italy; gerovetro@gmail.com (C.V.); maugericinzia@hotmail.com (C.M.); elixmauro@hotmail.it (E.M.); marinaparisi@hotmail.it (M.S.P.); diraimon@unict.it (F.D.R.); 3 Department of Scienze Mediche Chirurgiche e Tecnologie Avanzate “G.F. Ingrassia”, University of Catania, 95123 Catania, Italy; palumbo.gam@gmail.com (G.A.M.P.); sandrina.romano@gmail.com (A.R.); 4 Division of Anesthesiology and Intensive Care, A.O.U. “Policlinico-San Marco”, Via S. Sofia 78, 95123 Catania, Italy; ggiulius59@gmail.com

**Keywords:** *Saprochaete capitata*, immunocompromised patients, voriconazole, hematological malignancies, emergent and multiresistant micro-organisms

## Abstract

Infections occurring in immunocompromised patients after intensive chemotherapy are often difficult to eradicate and are capable of even being fatal. New emergent and dangerous drug-resistant micro-organisms are likely to appear in these specific scenarios. Clinical features mainly include progressive pneumonia, bacteriemia/fungemia, or extrapulmonary dissemination among infections. The treatment of these microorganisms is still an open challenge since there is a lack of clear treatment guidelines. Indeed, infections from these microorganisms can lead to a rapidly fatal clinical course in immunocompromised patients, especially those who have acute leukemia. We describe the case of a young patient with acute myeloid leukemia who contracted an infection from *Saprochaete capitata* during post-chemotherapy aplasia.

## 1. Case Presentation

**Figure 1:**
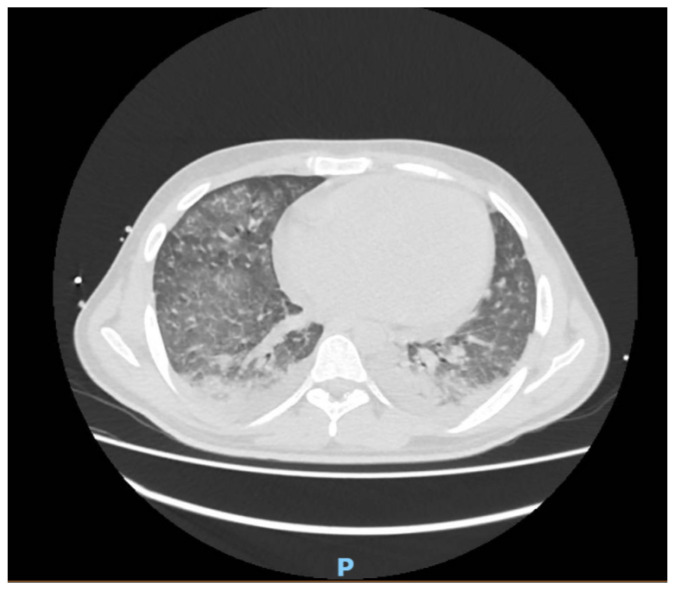
Evidence of widespread ground glass, perihiliar/interscissural fluid thickening, parenchymal atelectasis and pleural effusion at CT, as described in the text.

We report a case of a 23-year-old male patient who went to the emergency room with ongoing symptoms suggestive of infection (fever and asthenia) for one month. Because of the presence of the translocation t(8; 21) (q22; q22), *AML1/ETO* (RUNX1/RUNX1T1) [1], low-risk acute myeloid leukemia (AML) was diagnosed. At the time of hospital admission, the patient was treated empirically with the combination of amikacin and piperacillin/tazobactam for seven days until the fever resolved. Nevertheless, the site of infection had not been identified since both the chest X-ray and the urine, feces, and blood cultures from the peripheral vein were negative. According to current guidelines, the patient was treated with a “7 + 3” scheme based on the combination of daunorubicin 60 mg/m^2^ at days 1, 3, and 5 and cytarabine 100 mg/m^2^ by continuous intravenous infusion for 7 days [2]. To reduce the risk of fungal infection due to the profound aplasia that subjects treated with intensive chemotherapy develop, such as the “7 + 3” scheme, experience, our patient received anti-fungal prophylaxis with posaconazole 200 mg three times a day [3]. The clinical course was characterized by infectious complications during post-chemotherapy aplasia (Hb 7.1 g/dL, platelets 2000/mmc, white blood cells 200/mmc and 10/mmc neutrophils) with several daily fever peaks, at day +9 from treatment start. Clinically, in addition to the mentioned febrile episodes, the patient began to manifest an increasingly marked water retention and fluid accumulation at the subcutaneous area, with an appearance of edema with imprinted fovea all over the body, accompanied by diarrhea with several loose or watery stool, without any isolation of pathogens in stool cultures. Vital signs remained stationary (SpO2, blood pressure, and diuresis). On blood chemistry tests, C-reactive protein (CRP) increased (288.33 mg/L, normal values 0–5 mg/L). The value of 1-3-β-glucan (a polysaccharide essential for the formation of the fungal cell wall and directly proportional to fungal infection) was considerably elevated (220 pg/mL, normal values < 80 pg/mL) [4]. Chest computed tomography (CT) was performed to search for any source of infection, finding a widespread ground-glass appearance with a prevalence of perihiliar and interscissural fluid thickening and pleural effusion (anteroposterior diameter of about 25 mm) with associated parenchymal atelectasis predominant at the bases. As already clinically identified, a further radiological examination found diffuse edematous imbibition of the subcutaneous soft tissues in an anasarca manifestation (Figure 1). The patient was treated without success with different empirical antibiotic and antimycotic therapies (vancomycin, meropenem, linezolid, metronidazole, and caspofungin at standard dosages for 10 days).

The patient’s general condition rapidly deteriorated, both from the clinical point of view and in terms of laboratory test results. The fluid retention increased day by day, evolving in an anasarca state, and the patient’s weight increased from 60 kg to 70 kg. At the same time, the vital parameters worsened with hypotension (systolic blood pressure 80 mmHg), oliguria and pulmonary insufficiency—arterial pO_2_/fraction of inspired oxygen (FiO_2_) ratio 110, normal value > 300—which made it necessary to provide hemodynamic support with noradrenaline and respiratory support, initially with a Venturi’s mask, up to non-invasive ventilation (NIV) with FiO_2_ 60%. From the blood chemistry, multi-organ failure (MOF) was evident, with coagulation alterations, a rise in creatinine, hypoalbuminemia, and liver failure, with an increase in total bilirubin, aspartate aminotransferase, alanine aminotransferase, and alkaline phosphatase. Prohormone of brain natriuretic peptide (ProBNP) and troponin-I were also outside the normal ranges, at 1572 pg/mL (normal value < 100 pg/mL) and 45 µg/L (normal value < 10 µg/L), respectively. CRP remained elevated.

At this point (+20 days from chemotherapy start), from a peripheral vein and peripherally inserted central catheters (PICC) blood cultures, performed during a febrile peak, *Saprochaete capitata* was isolated (Table 1) by real-time PCR assay. It is an arthroconidia yeast-like filamentous fungi, related to the ascomycetous yeasts increasingly recognized as an emerging threat to immunocompromised patients [5].

Given the patient’s hemodynamic instability and severe clinical conditions, the patient was transferred to the intensive care unit for further monitoring of vital signs and to undergo life-saving supportive therapy.

For the fungal isolation described above and the removal and replacement of PICC, we based our approach on studies and a few clinical cases reported in the literature, where a severe prognosis was demonstrated in immunocompromised patients colonized by *Saprochaete capitata* [5]. We, therefore, decided to treat the patient with voriconazole 6 mg/kg loading dose, followed by a maintenance dose at 4 mg/kg twice a day. In addition, the patient underwent bronchoalveolar lavage (BAL) procedure, where the presence of *Klebsiella pneumoniae* was found. For this reason, antibiotic therapy was enhanced with the combination of fosfomycin 16 g/day divided into four administrations and ceftazidime/avibactam 2/0.5 g three times a day. After a slight but insufficient improvement in the patient’s clinical condition, we decided to intensify the antifungal therapy performed up to that point by combining voriconazole with amphotericin-B 5 mg/kg/day and by increasing the dose of voriconazole to 6 mg/kg twice a day, based on in vitro studies that show the efficacy of this combination against pathogens such as Aspergillus [6], plus a few case reports indicating the feasibility of this combination at standard dosages [5,7,8,9]. At the same time, we performed a re-evaluation of bone marrow aspirate, identifying post-chemotherapy aplasia without evidence of blasts. We performed therapy with subcutaneous colony-stimulating growth factors (G-CSF) [10].

After ten days of combination therapy, the patient’s symptoms progressively improved, reducing the generalized edema through fluid loss and febrile episodes, weaning from respiratory support with NIV, and adjusting vital parameters. This clinical improvement was accompanied by the progressive improvement of the altered blood chemistry laboratory indices, up to complete normalization, and, overall, contemporary total recovery of blood count values (Hb 11.1 g/dL, platelets 243,000/mmc, white blood cells 7890/mmc, and 5700/mmc neutrophils) (Table 2).

**Figure 2:**
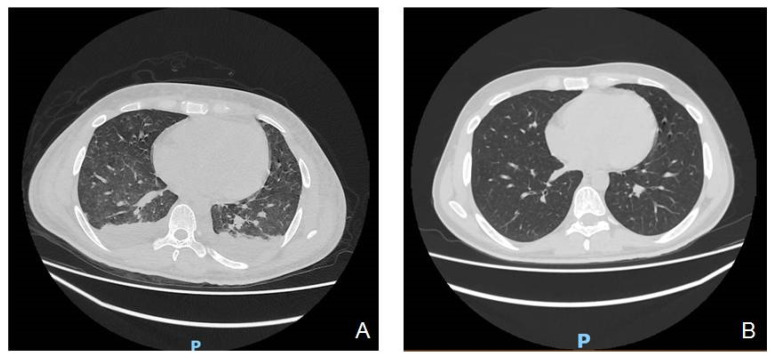
(**A**,**B**) CT scans of progressive improvement and disappearance of pulmonary fungal involvement were evaluated after 7 (**A**) and 20 days (**B**) from the start of antimycotic therapy. P, posterior of the chest.

The combination therapy with the two antifungals was carried out for a month, until the disappearance of the febrile episodes, normalization of 1-3-β-glucan values, and restoration of bilateral pulmonary physiological morphology, as demonstrated by consequential CT scans (Figure 2A,B).

Thus, after the resolution of *Saprochaete capitata* infection, the patient continued his therapeutic process, undergoing consolidation chemotherapy and maintaining a complete response.

## 2. Discussion

A prompt diagnosis of fungal infection is crucial in dealing with immunosuppressed hemato-oncological patients. In infections not responsive to the main antifungals, it is essential to look for uncommon infectious agents, e.g., microorganisms belonging to *Saprochaete* spp. [5], such as *Saprochaete clavata* (formerly *Geotrichum clavatum*) and *Saprochaete capitata* (formerly *Blastoschizomyces capitatus*, *Geotrichum capitatum* or *Magnusiomyces capitatus*), arthroconidia yeast-like filamentous fungi microbiologically and phylogenetically related to ascomycetous yeasts, and classified in the family *Dipodascaceae*, order *Saccharomycetales* [11,12].

*Saprochaete* spp. can be generally found in nature, especially in soil [13]. The majority of patients who develop invasive *Saprochaete* infection are probably initially colonized with the organism but without showing symptoms of infection [9]. Invasion through the respiratory or gastrointestinal tract is likely the entry site into the human host. Damage to the gastrointestinal mucosal barrier and chemotherapy-induced ulcerations might allow fungal translocation [14]. The transmission mode is not defined; however, the facts suggest that the man-to-man method of infection is possible, especially in immunocompromised patients, leading to environmental hospital contamination or device colonization [15,16,17].

In recent years, such microorganisms have played a pivotal role as emerging fungal pathogens responsible for life-threatening infections in immunocompromised patients, particularly in the setting of profound neutropenia. Deepening the studies in the literature, there are no clear guidelines on treatment, mainly due to the rarity of this infection and the lack of standardized antifungal breakpoints. In particular, these organisms appear to be intrinsically resistant to echinocandins and highly resistant to fluconazole [9,14]. A report in the literature tells of five cases of patients suffering from hematological diseases, affected by *Saprochaete capitata* and treated with various combinations of antifungals without finding the best therapy. The patients were affected by acute leukemia (four cases) and Burkitt’s lymphoma (one) in various lines of treatment (two in induction therapy and three with rescue schemes). *Saprochete capitata* was isolated from blood cultures or lungs (a single case, post-mortem finding). Clinical manifestations ranged from maculopapular rash, pancolitis, dyspnea, or septic shock. The two out of five surviving patients improved their clinical picture at the time of hematological recovery, increasing neutrophils at the end of the post-chemotherapy aplasia phase [18].

The European Society of Clinical Microbiology and Infectious Diseases (ESCMID) and the European Confederation of Medical Mycology (ECMM) guidelines recommend amphotericin B with or without flucytosine as initial therapy [19]. Our therapeutic choice was the association of voriconazole at loading doses (6 mg/kg two per day) and amphotericin-B 5 mg/kg/day, based on the few clinical studies—almost case reports—available in the literature [5,7,8,9,20], together with the close monitoring of laboratory and vital parameters. In these infections, the prompt recovery of blood counts seemed to be the only hope of survival in these patients, accompanied by the choice of therapy to guarantee a complete remission of the underlying hematological disease. The clinician’s goal should be to closely monitor vital signs with a possible transfer to the intensive care unit to undergo life-saving supportive therapy. As we have seen in the literature, existing antifungal drugs do not seem to guarantee an adequate response. Despite the guideline suggestions on choosing amphotericin-B as the first line of treatment, combination therapy appears to be the best therapeutic choice, as seen in this case with the addition of voriconazole. Finally, a pivotal role is played by possibly administering granulocytic growth factors to prompt the recovery of the organism’s cellular defenses.

Future studies are necessary to confirm the prospect of validity of this interesting therapeutic hypothesis, considering that these infections may further increase with the development of novel chemotherapeutic and immunosuppressive therapies.

## 3. Conclusions

The diagnosis of fungal or bacterial infection and the close monitoring of vital signs—with a possible transfer to the intensive care unit to undergo life-saving supportive therapy—are the critical points for the survival of these patients, underlining the importance of hematological recovery, which seems to be the only effective weapon in dealing with these rare cases of infection with emerging pathogens.

## Figures and Tables

**Table 1:** Antimycotic drugs and relative breakpoint for susceptibility in patient’s *Saprochete capitata*, as tested in the microbiology laboratory.

Antimycotic Drug	Breakpoints for Antifungal Susceptibility	Tested Breakpoint
Posaconazole	0.25	0.12–8
Amphotericin B	0.25	0.008–8
Fluconazole	4	0.015–8
Itraconazole	0.5	0.008–8
5-Fluorocytosine	0.12	0.06–64
Voriconazole	0.12	0.008–8
Caspofungin	>8	0.008–8
Anidulafungin	4	0.015–16
Micafungin	4	0.12–256

**Table 2:** Evidence of progressive laboratory improvement in blood chemistry tests, evaluated at baseline, and at day +5, +10 and +20 from start of antimycotic therapy. INR, international normalized ratio; AST, aspartate transaminase; LDH, lactate dehydrogenase; CRP, C-reactive protein.

	Baseline	Day +5	Day +10	Day +20	Normal Value
INR	1.8	1.77	1.23	1.13	0.8–1.2
Creatinine	3.54	1.89	1.00	0.69	0.67–1.17 mg/dL
Albumin	1.8	2.37	3.72	4.1	3.5–5.2 g/dL
Total bilirubin	2.14	1.82	1.67	0.94	0.3–1.2 mg/dL
AST	127	86	30	34	0–30 U/L
LDH	495	398	290	151	0–248 U/L
CRP	384	250	53	34	0–5 mg/L
Cholinesterase	<1000	<1000	<1000	2950	4620–11,500 U/L

## References

[1] Döhner H., Estey E., Grimwade D., Amadori S., Appelbaum F.R., Büchner T., Dombret H., Ebert B.L., Fenaux P., Larson R.A. (2017). Diagnosis and management of AML in adults: 2017 ELN recommendations from an international expert panel. Blood.

[2] Wiernik P., Banks P., Case D.J., Arlin Z., Periman P., Todd M., Ritch P., Enck R., Weitberg A. (1992). Cytarabine plus idarubicin or daunorubicin as induction and consolidation therapy for previously untreated adult patients with acute myeloid leukemia. Blood.

[3] Girmenia C., Frustaci A.M., Gentile G., Minotti C., Cartoni C., Capria S., Trisolini S.M., Matturro A., Loglisci G., Latagliata R. (2012). Posaconazole prophylaxis during front-line chemotherapy of acute myeloid leukemia: A single-center, real-life experience. Haematologica.

[4] Lamoth F., Cruciani M., Mengoli C., Castagnola E., Lortholary O., Richardson M., Marchetti O. (2012). β-glucan antigenemia assay for the diagnosis of invasive fungal infections in patients with hematological malignancies: A systematic review and meta-analysis of cohort studies from the third European Conference on Infections in Leukemia (ECIL-3). Clin. Infect. Dis..

[5] El Zein S., Hindy J.-R., Kanj S.S. (2020). Invasive Saprochaete Infections: An Emerging Threat to Immunocompromised Patients. Pathogens.

[6] Elefanti A., Mouton J.W., Verweij P.E., Tsakris A., Zerva L., Meletiadis J. (2013). Amphotericin B- and Voriconazole-Echinocandin Combinations against Aspergillus spp.: Effect of Serum on Inhibitory and Fungicidal Interactions. Antimicrob. Agents Chemother..

[7] Favre S., Rougeron A., Pérard N., Levoir L., Pérard B., Milpied N., Accoceberry I., Gabriel F., Vigouroux S. (2016). Saprochaete clavata invasive infection in a patient with severe aplastic anemia: Efficacy of voriconazole and liposomal amphotericin B with adjuvant granulocyte transfusions before neutrophil recovery following allogeneic bone marrow transplantation. Med. Mycol. Case Rep..

[8] Lajolo C., Rupe C., Schiavelli A., Gioco G., Metafuni E., Contaldo M., Sica S. (2021). Saprochaete clavata Infection in Immunosuppressed Patients: Systematic Review of Cases and Report of the First Oral Manifestation, Focusing on Differential Diagnosis. Int. J. Environ. Res. Public Health.

[9] Buchta V., Bolehovská R., Hovorková E., Cornely O.A., Seidel D., Žák P. (2019). Saprochaete clavata Invasive Infections—A New Threat to Hematological-Oncological Patients. Front. Microbiol..

[10] Vacek A., Hofer M., Holá J., Weiterová L., Štreitová D., Svoboda J. (2007). The role of G-CSF and IL-6 in the granulopoiesis-stimulating activity of murine blood serum induced by perorally administered ultrafiltered pig leukocyte extract, IMUNOR^®^. Int. Immunopharmacol..

[11] Hoog G.S., Smith M., Guého E. (1986). A Revision of the Genus Geotrichum and Its Teleomorphs.

[12] Desnos-Ollivier M., Blanc C., Garcia-Hermoso D., Hoinard D., Alanio A., Dromer F. (2014). Misidentification of saprochaete clavata as magnusiomyces capitatus in clinical isolates: Utility of internal transcribed spacer sequencing and matrix-assisted laser desorption ionization-time of flight mass spectrometry and importance of reliable databases. J. Clin. Microbiol..

[13] Pamidimukkala U., Kancharla A., Sudhaharan S., Gundeti S., Mandarapu S., Nagalla V.K., Raju S.B., Karanam S.D. (2017). Isolation of the Rare Opportunistic Yeast Saprochaete capitata from Clinical Samples-Experience from a Tertiary Care Hospital in Southern India and a Brief Review of the Literature. J. Clin. Diagn. Res..

[14] Lo Cascio G., Vincenzi M., Soldani F., De Carolis E., Maccacaro L., Sorrentino A., Nadali G., Cesaro S., Sommavilla M., Niero V. (2020). Outbreak of Saprochaete clavata Sepsis in Hematology Patients: Combined Use of MALDI-TOF and Sequencing Strategy to Identify and Correlate the Episodes. Front. Microbiol..

[15] Vaux S., Criscuolo A., Desnos-Ollivier M., Diancourt L., Tarnaud C., Vandenbogaert M., Brisse S., Coignard B., Dromer F., Group T.G.I. (2014). Multicenter Outbreak of Infections by Saprochaete clavata, an Unrecognized Opportunistic Fungal Pathogen. MBio.

[16] Menu E., Criscuolo A., Desnos-Ollivier M., Cassagne C., D’Incan E., Furst S., Ranque S., Berger P., Dromer F. (2020). Saprochaete clavata Outbreak Infecting Cancer Center through Dishwasher. Emerg. Infect. Dis..

[17] Kaplan E., Al-Hatmi A.M.S., Ilkit M., van den Ende A.H.G.G., Hagen F., Meis J.F., de Hoog G.S. (2018). Molecular Diagnostics of Arthroconidial Yeasts, Frequent Pulmonary Opportunists. J. Clin. Microbiol..

[18] Garcia-Ruiz J.C., Lopez-Soria L., Olazabal I., Amutio E., Arrieta-Aguirre I., Velasco-Benito V., Pontón J., Moragues M.-D. (2013). Invasive infections caused by Saprochaete capitata in patients with haematological malignancies: Report of five cases and review of the antifungal therapy. Rev. Iberoam. Micol..

[19] Arendup M.C., Boekhout T., Akova M., Meis J.F., Cornely O.A., Lortholary O. (2014). ESCMID and ECMM joint clinical guidelines for the diagnosis and management of rare invasive yeast infections. Clin. Microbiol. Infect..

[20] Li S.S., Tang X.Y., Ni S.L., Yang N.-B., Lu M.-Q. (2016). Voriconazole combined with low-dose amphotericin B liposome for treatment of cryptococcal meningitis. Infect. Dis..

